# Phase 2 study of cabozantinib (XL184) with nivolumab and ipilimumab for the treatment of poorly differentiated neuroendocrine carcinomas (ETCTN10315)

**DOI:** 10.1093/oncolo/oyag140

**Published:** 2026-04-10

**Authors:** Adel Mandl, Ruizhe Chen, Satya Das, Jonathan R Strosberg, Anup Kasi, Bhavana Konda, Daneng Li, Timothy F Burns, Aman Chauhan, Kristen K Ciombor, Farshid Dayyani, Tanya Dorff, James P Ohr, Bhaumik B Patel, Vineeth Sukrithan, Heloisa P Soares, Kang Chen, Stephen V Liu, Anteneh A Tesfaye, Michael A Carducci, Jan H Beumer

**Affiliations:** Department of Oncology, Johns Hopkins University School of Medicine and Sidney Kimmel Comprehensive Cancer Center at Johns Hopkins, Baltimore, MD, USA; Department of Oncology, Johns Hopkins University School of Medicine and Sidney Kimmel Comprehensive Cancer Center at Johns Hopkins, Baltimore, MD, USA; Vanderbilt University Medical Center, Nashville, TN, USA; H. Lee Moffitt Cancer Center & Research Institute, Tampa, FL, USA; University of Kansas Cancer Center, Kansas City, KS, USA; The Ohio State University Comprehensive Cancer Center—James Cancer Hospital and Solove Research Institute, Columbus, OH, USA; City of Hope National Medical Center, Duarte, CA, USA; UPMC Hillman Cancer Center, Pittsburgh, PA, USA; Sylvester Comprehensive Cancer Center, University of Miami, FL, USA; Vanderbilt University Medical Center, Nashville, TN, USA; Chao Family Comprehensive Cancer Center, University of California Irvine, Orange, CA, USA; City of Hope National Medical Center, Duarte, CA, USA; UPMC Hillman Cancer Center, Pittsburgh, PA, USA; VCU Massey Cancer Center, Virginia Commonwealth University, Richmond, VA, USA; The Ohio State University Comprehensive Cancer Center—James Cancer Hospital and Solove Research Institute, Columbus, OH, USA; Huntsman Cancer Institute, University of Utah, Salt Lake City, UT, USA; Barbara Ann Karmanos Cancer Institute, Wayne State University School of Medicine, Obstetrics and Gynecology, Detroit, MI, USA; Georgetown Lombardi Comprehensive Cancer Center, Georgetown University, Washington, DC, USA; Georgetown Lombardi Comprehensive Cancer Center, Georgetown University, Washington, DC, USA; Department of Oncology, Johns Hopkins University School of Medicine and Sidney Kimmel Comprehensive Cancer Center at Johns Hopkins, Baltimore, MD, USA; Department of Oncology, Johns Hopkins University School of Medicine and Sidney Kimmel Comprehensive Cancer Center at Johns Hopkins, Baltimore, MD, USA

**Keywords:** neuroendocrine carcinoma, tyrosine kinase inhibitor, immunotherapy, checkpoint inhibitors, cabozantinib, ipilimumab, nivolumab

## Abstract

**Background:**

Poorly differentiated neuroendocrine carcinomas (NECs) are aggressive malignancies with limited treatment options. The low activity of immune checkpoint inhibitors (ICI) is in part due to an immunosuppressive tumor microenvironment. We hypothesized that adding cabozantinib to dual ICIs may improve clinical outcomes for patients.

**Methods:**

Patients with advanced or metastatic, poorly differentiated NECs, excluding small cell lung and Merkel cell carcinoma, who progressed on first-line systemic therapy enrolled on this Simon’s two-stage trial. Patients received cabozantinib 40 mg daily, 3 mg/kg nivolumab plus 1 mg/kg ipilimumab every 3 weeks for four cycles, followed by maintenance cabozantinib nivolumab. The primary endpoint was ORR.

**Results:**

Of 17 patients enrolled, 16 were evaluable. ORR was 12.5% (2/16; 95%CI: 1.6%-38.3%), with 2 durable (27 and 17 months). Median PFS was 2.7 months (95%CI: 1.8-5.6 months). Grade 3-4 treatment-related adverse events occurred in 9 patients (56%). The most frequent grade ≥3 toxicities were elevated liver enzymes, fatigue, and hypertension. Only one patient discontinued treatment due to toxicity. The study was terminated after the first stage, as it did not meet the threshold for proceeding to stage 2.

**Conclusion:**

Toxicities were consistent with the known safety profiles of cabozantinib and dual immune checkpoint blockade. Long-term responses were observed in a small subset of patients, but the study did not meet the prespecified efficacy threshold for continued accrual.

**ClinicalTrials.gov identifier:**

NCT04079712

Lessons learned

- In this phase II trial, cabozantinib with nivolumab and ipilimumab demonstrated a manageable safety profile and limited clinical activity in patients with advanced and metastatic poorly differentiated neuroendocrine carcinomas.
- Notably, the two responders achieved durable partial responses, highlighting that, although rare, meaningful long-term benefit is possible in some patients.
- Despite manageable safety and rare durable responses, the limited overall activity does not support further development of this regimen in poorly differentiated NECs.

## Trial information

**Table:** 

**Trial information**	
Disease	Advanced/metastatic poorly differentiated neuroendocrine neoplasms with the exception of Merkel cell cancer and small cell lung cancer
Stage of Disease/Treatment	III-IV
Prior Therapy	1 prior regimen
Type of Study	Phase II, single-arm, open label clinical trial
Primary Endpoints	Overall response rate (CR+PR) based on RECIST v1.1 criteria^1^
Secondary Endpoints	Progression-free survival, disease control rate, duration of response, safety and tolerability
Additional Details of Endpoints or Study Design This Phase II study used a Simon’s two-stage minimax design^2^ with a primary endpoint of overall response rate (CR+PR) by RECIST v1.1^1^. The study hypothesized that cabozantinib, nivolumab, and ipilimumab would increase the ORR in poorly differentiated NECs after 1 prior therapy from 45% to 70%, with 90% power and a 10% type I error. Responses had to be confirmed within 6 months. Both all-registered and response-evaluable populations (≥1 dose, baseline measurable disease, ≥1 post-baseline assessment) were analyzed, with rates summarized using point estimates and Wilson-type 95% CIs^3^. An interim analysis assessed both efficacy and safety: if ≥7 of the first 15 patients responded and ≤4 had significant treatment delays/discontinuations due to toxicity, accrual would continue to stage two. If ≥5 patients had such toxicity despite adequate response, accrual was paused pending CTEP review. Morphologic subtypes and primary sites were collected descriptively, but no stratification was applied.	

## Drug information

Following the initial four cycles of triplet therapy, patients continued maintenance treatment with cabozantinib and nivolumab until disease progression or unacceptable toxicity.

**Table:** 

**Cabozantinib information**	
Generic/Working name	Cabozantinib (XL184)
Company Name	Exelixis
Drug Type	Targeted therapy
Drug Class	Tyrosine kinase inhibitor (TKI) of MET, VEGFR, AXL, and others^4^
Dose	40 mg orally once daily
Route	PO
Schedule of Administration: daily, continuous	

**Table:** 

**Nivolumab information**	
Generic/Working name	Nivolumab
Company Name	Bristol Myers Squibb
Drug Type	Immunotherapy
Drug Class	PD-1 checkpoint inhibitor (IgG4 monoclonal antibody)^5^
Dose	Cycles 1-4: 3 mg/kg Cycles 5+: 480 mg
Route	IV
Schedule of Administration: Cycles 1-4: 3 mg/kg on Day 1 of cycles, every 21 days Cycles 5+: 480 mg on Day 1 of cycles, every 28 days	

**Table:** 

**Ipilimumab information**	
Generic/Working name	Ipilimumab
Company Name	Bristol Myers Squibb
Drug Type	Immunotherapy
Drug Class	CTLA-4 checkpoint inhibitor (IgG1 monoclonal antibody)^6^
Dose	1 mg/kg (4 total doses)
Route	IV
Schedule of Administration: 1 mg/kg on Day 1 of cycles 1-4, every 21 days	

## Patient characteristics

**Table:** 

**Patient characteristics**	
Number of Patients, Male	12 (75%)
Number of Patients, Female	4 (25%)
Age: median	64 years
Stage	Stage III: 1 Stage IV: 15
Number of Prior Systemic Therapies: Median (Range)	1 prior treatment: 13 2 prior treatments: 3*
Performance Status: ECOG 0 or 1	0-4 1-12
Performance Status: ECOG 2 or above	0
Cancer types Histologic Subtypes per pathology report	GI: 12, prostate: 1, nasopharynx: 1, lung: 1, unknown: 1 Small cell NEC: 5 Large cell NEC: 6 Mixed NEC: 1 Poorly differentiated NEC (unknown subtype): 4

* Eligibility required failure of one prior line of systemic therapy in the advanced/metastatic setting. Two patients received platinum-etoposide twice, with retreatment at progression. This was counted as two prior therapies. A third patient had received prior adjuvant platinum-etoposide followed by CAPTEM at recurrence, which represents one prior line in the metastatic setting.

Baseline demographic, clinical, and disease characteristics are summarized in Table 1.

**Table 1:** 

Baseline demographic, clinical, and disease characteristics (*N* = 16)	
Characteristics	*n* (%)
Sex	
Female	4 (25%)
Male	12 (75%)
Race	
Asian	1 (6.3%)
Black or African American	1 (6.3%)
White	14 (88%)
Ethnicity	
Hispanic or Latino	1 (6.3%)
Not Hispanic or Latino	14 (88%)
Not Reported	1 (6.3%)
Age (yrs)	
Mean Median SD (Q1, Q3)	62 64 12 (55, 74)
Weight (kg)	
Mean Median SD (Q1, Q3)	86 86 20 (73, 101)
Height (cm)	
Mean Median SD (Q1, Q3)	174 176 8 (168, 179)
BSA	
Mean Median SD (Q1, Q3)	2.01 2.03 0.27 (1.86, 2.19)
ECOG PS	
0	4 (25%)
1	12 (75%)
Primary Disease Site	
Adrenal Gland	1 (6.3%)
Cecum	1 (6.3%)
Unknown GI Origin	1 (6.3%)
Liver	1 (6.3%)
Lung	1 (6.3%)
Unknown	1 (6.3%)
Nasopharynx	1 (6.3%)
Pancreas	7 (43.7%)
Rectum	2 (12.5%)
Gastric	1 (6.3%)
Histology	
Small cell NEC	5 (31.3%)
Large cell NEC	6 (37.5%)
Mixed NEC	1 (6.3%)
Poorly differentiated NEC (unknown subtype)	4 (25.1%)
Prior Surgery	
No	14 (88%)
Yes	2 (13%)
Prior Chemo	
Carbo-etoposide	13 (81%)
Cisplatin-etoposide	2 (13%)
Etoposide	1 (6.3%)
Prior RT	
No	10 (63%)
Yes	6 (38%)

Values are presented as n (%) unless otherwise noted; continuous variables shown as mean, median, SD, (range).

## Primary assessment method

**Table:** 

Primary assessment method	
Title	Overall response rate (ORR)
Number of Patients Screened	17
Number of Patients Enrolled	17
Number of Patients Evaluable for Toxicity	16
Number of Patients Evaluated for Efficacy	16
Evaluation Method	RECIST 1.1
Outcome Notes Response Assessment CR *n* = 0 (0%) Response Assessment PR *n* = 2 (12.5%) Response Assessment SD *n* = 8 (50%) Response Assessment PD *n* = 6 (37.5%)	

## Serious adverse events (SAEs)

**Table:** 

Serious adverse events		
**Dose level**	**Adverse event(s), grade**	**Attribution**
1	Hepatic failure, gr 4	Possible
1	Fall, gr 2	Unlikely
1	Hepatic failure (DILI, immune-related), gr 4	Related
1	Pericardial effusion, gr 3	Possible
1	Thromboembolic event, gr 4	Possible
1	Alanine aminotransferase increased, gr 3	Related
1	Aspartate aminotransferase increased, gr 3	Related
1	Acute kidney injury, gr 3	Possible
1	Cholangitis, gr 3	Unrelated
1	Non-cardiac chest pain, gr 3	Unlikely
1	Stoma site infection, gr 3	Unlikely
1	Tracheitis, gr 3	Unlikely

## Toxicity profile—grade 3–4 events

**Table:** 

Toxicity profile—Grade 3-4 events		
Treatment-related adverse event	All grades *n* (%)	Grade 3-4 *n* (%)
ALT increase	11 (69)	3 (19)
AST increase	10 (63)	3 (19)
Fatigue	10 (63)	3 (19)
Thrombocytopenia	7 (44)	1 (6)
Hyponatremia	6 (38)	1 (6)
Hypertension	4 (25)	2 (13)
Lipase increase	4 (25)	1 (6)
Hyperbilirubinemia	3 (19)	1 (6)
Lymphopenia	3 (19)	1 (6)
Muscle weakness	2 (13)	1 (6)
Hepatic failure	2 (13)	2 (13)
Appendicitis	1 (6)	1 (6)
Fibrinogen decreased	1 (6)	1 (6)
Hearing impaired	1 (6)	1 (6)
Hyperglycemia	1 (6)	1 (6)
Myalgia	1 (6)	1 (6)
Pain	1 (6)	1 (6)
Pericardial effusion	1 (6)	1 (6)
Thromboembolic event	1 (6)	1 (6)

## Discussion

This single-arm phase II study evaluated cabozantinib, ipilimumab, and nivolumab in patients with poorly differentiated NEC, a rare and aggressive malignancy with few effective treatment options. In 16 evaluable patients, the overall response rate (ORR) was 12.5%, including two durable partial responses lasting 27 and 17 months. Most cases of stable disease were short-lived, with only one patient maintaining stability beyond 12 months. Median PFS was 2.7 months (Figure S1) and OS 18.6 months (Figure S2). Taken together, these results highlight limited overall efficacy, with a small number of patients experiencing prolonged benefit. Treatment exposure and clinical outcomes are summarized in Table 2. The safety profile was consistent with the known toxicities of cabozantinib and dual immune checkpoint blockade.^7^^,^^8^

**Table 2.** 

Treatment exposure and clinical outcomes	
Total cycles, mean median SD (Q1, Q3)	8 4 11 (2, 7)
Nivolumab cycles	8 3 11 (2, 7)
Ipilimumab cycles	3 3 1 (2, 4)
Cabozantinib cycles	7 3 9 (2, 6)
Reason for treatment discontinuation, n (%)	
Adverse event	1 (6.3%)
Physician discretion	1 (6.3%)
Progressive disease	13 (81%)
Poor performance status	1 (6.3%)
Best Response, n (%)	
PR	2 (13%)
PD	7 (44%)
SD	7 (44%)

Abbreviations: CR, complete response; PR, partial response; SD, stable disease; PD, progressive disease.

NECs are rare, biologically heterogeneous tumors, which have limited the conduct of prospective studies. Standard first-line platinum-etoposide has an ORR of ∼31%, median PFS of 4 months, and median OS of 10-12 months.^9^ Second-line chemotherapy has ORR rarely exceeding 10%-15% and median survival is around 7-8 months.^10^ To improve this, several prospective studies have tested dual immune checkpoint blockade with heterogeneous outcomes. In the DART (SWOG S1609) trial, (second-line) nivolumab plus ipilimumab achieved an ORR of 26% (*N* = 19), with median PFS of 2.0 months and OS of 8.7 months.^11^ Similarly, the CA209-538 study observed a 24% ORR (*N* = 29) overall (31% in high-grade subgroups), with median PFS 4.8 months and OS 14.8 months.^12^ By contrast, the NIPINEC trial reported more modest efficacy, with ORRs of 14.9% (*N *= 185) for the combination and median PFS and OS of 1.9 and 5.8 months, respectively.^13^ Collectively, these studies highlight the heterogeneous and overall lackluster activity of dual checkpoint blockade in NECs.

The combination of cabozantinib with immune checkpoint inhibitors is supported by strong biologic rationale and clinical data.^7^ Cabozantinib is a multi-target tyrosine kinase inhibitor (VEGFR2, MET, AXL, RET, and TAM kinases) that not only suppresses tumor angiogenesis and oncogenic signaling but also reshapes the tumor microenvironment by decreasing immunosuppressive regulatory T cells and myeloid-derived suppressor cells, enhancing T-cell infiltration, and normalizing tumor vasculature to improve immune access.^4^ While cabozantinib has demonstrated efficacy in well-differentiated NETs in the Phase III CABINET trial supporting labeling,^14^ its role in poorly differentiated NEC remains uncertain. In our study, overall activity was limited, although two patients achieved partial responses lasting 27 and 17 months. These findings should be interpreted cautiously, as the single-arm design does not allow the contribution of cabozantinib to be definitively determined. Notably, the observed response rate and the presence of durable responders are also consistent with prior studies of dual immune checkpoint blockade alone.^13^

Across prior studies, response to dual immune checkpoint blockade has been influenced by tumor grade and site of origin. Activity appears greatest in high-grade tumors,^15^ with particularly encouraging signals in high-grade pancreatic NENs and atypical bronchial carcinoids.^12^ Median Ki-67 indices are typically very high, but no reproducible threshold has emerged to stratify benefit.^11^

Our 17-month responder had gastric small cell NEC with high Ki-67 (90%) and 5% PD-L1 expression. Molecular profiling revealed loss-of-function alterations in RHOA and TP53. Microsatellite instability testing was negative. Despite these high-risk molecular features^16^^,^^17^ and the typically poor prognosis of high-grade GI NECs,^18^ this patient remained on therapy for over 25 months.

Our 27-month responder had treatment-emergent neuroendocrine prostate cancer with high-grade histology (Ki-67 90%) and adrenal metastasis following prior prostate-directed and systemic therapies. Enrolled after only a brief response to carboplatin-etoposide, he received study treatment for 36 months. Upon oligoprogression, he underwent SBRT to the adrenal lesion and has continued cabozantinib and nivolumab beyond trial completion with ongoing disease control to date. Germline testing revealed a pathogenic MUTYH variant, which may be relevant to treatment response in the context of DNA repair alterations.^19^

Our patient with stable disease lasting 14 months had stage IV large cell pancreatic NEC with lower Ki-67 (35%). Microsatellite instability testing was negative. Molecular profiling showed pathogenic copy number loss of MTAP and CDKN2A, as well as a pathogenic frameshift mutation in DAXX and a missense loss-of-function mutation in CREBBP. Despite these adverse molecular features,^20^ the patient’s disease remained stable for over a year. These cases underscore the potential for rare but durable benefit with immunotherapy-based strategies and highlight the biological heterogeneity of NECs and the importance of incorporating molecular profiling to identify yet biologically undefined subsets of patients that may derive disproportionate benefit.

We report a median overall survival of 18.6 months in this cohort. While this is longer than that reported in prior studies of dual checkpoint blockade in NEC, the result should be interpreted cautiously. Several limitations must be acknowledged. The trial was terminated early after failing to meet the stage 1 efficacy threshold (defined as response in at least 7 out of 15), resulting in a small sample size that limits statistical power and precludes meaningful conclusions. The nonrandomized design further complicates interpretation, preventing attribution of benefit to any single agent within the regimen. In addition, overall survival may be influenced by the small number of long-term responders, lack of detailed data on subsequent therapies, and the inherent limitations of cross-trial comparisons. Finally, the statistical design warrants consideration. The Simon minimax design tested a null hypothesis of 45% ORR versus an alternative of 70%, with a 6-month endpoint. This high bar was set during a period of early enthusiasm for dual checkpoint blockade, when preliminary reports suggested higher activity than what was later confirmed in larger studies. Subsequent data from DART, NIPINEC, and CA209-538 have consistently shown ORRs in the 15%-30% range, making the null of 45% overambitious, while the toxicity of these combinations is an important consideration. As a result, the trial did not reach its interim target despite the clinically meaningful and durable responses observed.

This study demonstrates that VEGFR inhibition combined with dual checkpoint blockade can yield durable benefit in a subset of patients with NEC. Future studies should prioritize biomarker development to refine patient selection and better identify those most likely to benefit from immunotherapy-based combinations.

## Supplementary Material

oyag140_Supplementary_Data

## Data Availability

The data generated in this study are publicly available in ClinicalTrials.gov (NCT04079712).

## CRediT

Adel Mandl (Investigation), Ruizhe Chen (Investigation), Satya Das (Investigation), Jonathan Strosberg (Investigation), Anup Kasi (Investigation), Bhavana Konda (Investigation), Daneng Li (Investigation), Timothy Burns (Investigation), Aman Chauhan (Investigation), Kristen Ciombor (Investigation), Farshid Dayyani (Investigation), Tanya Dorff (Investigation), James Ohr (Investigation), Bhaumik Patel (Investigation), Vineeth Sukrithan (Investigation), Heloisa Soares (Investigation), Kang Chen (Investigation), Stephen V Liu (Investigation), Anteneh A Tesfaye (Investigation), and Michael A Carducci (Investigation), Jan Beumer (Investigation).
