# Supplementary material for: Phase 2 study of cabozantinib (XL184) with nivolumab and ipilimumab for the treatment of poorly differentiated neuroendocrine carcinomas (ETCTN10315)
Source: Oncologist. 2026 Apr 10;31(6):oyag140. doi: 10.1093/oncolo/oyag140 (PMC13181262; doi:10.1093/oncolo/oyag140)
Supplement: oyag140_Supplementary_Data [file oyag140_supplementary_data.pdf]

SUPPLEMENT

Phase 2 Study of Cabozantinib (XL184) with Nivolumab and Ipilimumab for the Treatment of Poorly Differentiated Neuroendocrine Carcinomas (ETCTN10315)

Adel Mandl[1], Ruizhe Chen[1], Satya Das[2,a], Jonathan R. Strosberg[3], Anup Kasi[4], Bhavana Konda[5], Daneng Li[6], Timothy F. Burns[7,b], Aman Chauhan[8], Kristen K. Ciombor[2], Farshid Dayyani[9], Tanya Dorff[6], James P. Ohr[7], Bhaumik B. Patel[10,c], Vineeth Sukrithan[5], Heloisa P. Soares[11], Kang Chen[12], Stephen V. Liu[13], Anteneh A. Tesfaye[13,d], Michael A. Carducci[1], Jan H. Beumer[1]

[1]Department of Oncology, Johns Hopkins University School of Medicine and Sidney Kimmel Comprehensive Cancer Center at Johns Hopkins, Baltimore, MD, USA
[2]Vanderbilt University Medical Center, Nashville, TN, USA
[3]H. Lee Moffitt Cancer Center & Research Institute, Tampa, FL, USA
[4]University of Kansas Cancer Center, Kansas City, KS, USA
[5]The Ohio State University Comprehensive Cancer Center – James Cancer Hospital and Solove Research Institute, Columbus, OH, USA
[6]City of Hope National Medical Center, Duarte, CA, USA
[7]UPMC Hillman Cancer Center, Pittsburgh, PA, USA
[8]Sylvester Comprehensive Cancer Center, University of Miami, FL, USA
[9]Chao Family Comprehensive Cancer Center, University of California Irvine, Orange, CA, USA
[10]VCU Massey Cancer Center, Virginia Commonwealth University, Richmond, VA, USA
[11]Huntsman Cancer Institute, University of Utah, Salt Lake City, UT, USA
[12]Barbara Ann Karmanos Cancer Institute, Wayne State University School of Medicine, Obstetrics and Gynecology, Detroit, MI, USA
[13]Georgetown Lombardi Comprehensive Cancer Center, Georgetown University, Washington, DC, USA

Supplementary methods:

Detailed Eligibility Criteria:
Eligible patients were required to have histologically confirmed, metastatic, poorly differentiated neuroendocrine carcinomas (NECs) as defined by the 2018 WHO classification, excluding small cell lung cancer and Merkel cell carcinoma. Variants of poorly differentiated NEC, including small cell, large cell, and mixed subtypes, were permitted. Patients were required to have measurable disease per RECIST v1.1, to have received exactly one prior line of systemic therapy, and to have safely biopsiable lesions with willingness to undergo paired tumor biopsies and baseline blood collection. Prior systemic therapy had to be completed at least four weeks before initiation of study treatment. Patients were required to be ≥18 years of age, have an ECOG performance status of 0–2, and adequate organ and marrow function, including leukocytes ≥3,000/µL, ANC ≥1,500/µL (without G-CSF support), hemoglobin ≥9 g/dL, platelets ≥100,000/µL, total bilirubin ≤1.5 × ULN (≤3 × ULN for Gilbert's), AST/ALT ≤3 × ULN, creatinine ≤ ULN or CrCl ≥50 mL/min/1.73 m², albumin ≥2.8 g/dL, and normal electrolytes and coagulation parameters. Patients with HIV, HBV, or HCV infection were eligible if viral load was undetectable on appropriate therapy. Patients with treated brain metastases were eligible provided there was no evidence of progression, steroid therapy had been discontinued ≥4 weeks, and ≥3 months had elapsed since brain surgery. Patients with prior or concurrent malignancies were permitted if the natural history or treatment was not expected to interfere with study assessments.

Women of childbearing potential and men with partners of childbearing potential were required to use effective contraception for the duration of treatment and for 5 and 7 months, respectively, after the last dose of study therapy. Women were required to have a negative pregnancy test within 24 hours before treatment initiation, and breastfeeding was not permitted.

Key exclusion criteria included requirement for systemic corticosteroids ≥10 mg/day prednisone equivalent (except physiologic replacement), prior therapy with PD-1/PD-L1/PD-L2 or CTLA-4 inhibitors, prior cabozantinib or other MET-targeting therapies, prior radiation or chemotherapy within 4 weeks of enrollment, or unresolved >grade 1 toxicities from previous therapy. Additional exclusions were uncontrolled cardiovascular disease (including NYHA class III/IV heart failure, unstable angina, recent MI or stroke, uncontrolled hypertension, or recent thromboembolic events), QTcF >500 msec, untreated or progressive brain or leptomeningeal metastases, recent major surgery (<4 weeks), active or high-risk gastrointestinal disorders (including bowel perforation, fistula, obstruction, or active IBD), significant hemorrhage within 12 weeks, or cavitating pulmonary lesions. Patients requiring therapeutic anticoagulation with warfarin, DOACs, or platelet inhibitors were excluded, although prophylactic aspirin or stable LMWH was permitted under specified conditions. Patients with active autoimmune disease requiring systemic therapy were excluded, with exceptions for controlled endocrinopathies, vitiligo, psoriasis treated with topical therapy, and other clinically stable conditions. Pregnant or breastfeeding women, and patients with psychiatric or social conditions precluding adherence, were not eligible.

Statistical Considerations:
This was a phase II, two-stage Simon minimax design evaluating the combination of

cabozantinib, nivolumab, and ipilimumab in patients with poorly differentiated neuroendocrine carcinomas (NECs) who had received at least one prior line of systemic therapy. The primary endpoint was overall response rate (ORR), defined as the proportion of patients achieving a confirmed complete response (CR) or partial response (PR) by RECIST v1.1 within 6 months of treatment initiation. The null hypothesis assumed a true response rate of 45%, whereas the alternative hypothesis was 70%, with a type I error of 0.10 and 90% power ($\beta = 0.10$). ORR was summarized with point estimates and two-sided Wilson confidence intervals for both the registered and response-evaluable (r-e) populations, with the latter defined as patients who received at least one dose of study drug, had measurable disease at baseline, and underwent at least one post-baseline disease assessment.

Stage 1 of the design planned to enroll 18 patients to obtain 15 evaluable patients. If seven or more patients achieved CR or PR within 6 months and no more than four patients experienced treatment delays or discontinuations due to toxicity, the study would proceed to Stage 2, which planned to accrue an additional 10 evaluable patients. If the first stage response target was not reached, the study would be terminated for futility. The overall sample size was 25 evaluable patients, with a maximum of 30 allowed to account for ineligibility. Under the null hypothesis, the design had an average sample number of 19.1 patients and a probability of early termination of 45%, with accrual estimated at approximately one patient per month.

Secondary endpoints included progression-free survival (PFS), overall survival (OS), disease control rate (DCR), duration of response (DOR), and safety. PFS and OS were analyzed using the Kaplan–Meier method, with medians and 95% confidence intervals estimated from the life table. DCR was defined as the proportion of patients achieving CR, PR, or stable disease, while DOR was measured from the first documentation of CR or PR until progression or death. Safety was assessed in all treated patients using CTCAE v5.0, with binary endpoints summarized as proportions with Wilson confidence intervals and toxicity distributions described by frequency. Exploratory correlative studies included paired biopsies assessed by immunohistochemistry and flow cytometry to evaluate intratumoral immune cells, tumor-associated macrophages, and myeloid-derived suppressor cells. For these endpoints, descriptive statistics (mean, standard deviation, median, interquartile range, and range) were reported, and boxplots and dot plots were generated to illustrate distributions. Analyses were performed separately for pretreatment samples, on-treatment samples, and pre/post-treatment differences.

All patients who received study treatment were evaluable for toxicity, and all eligible patients were included in the main efficacy analysis, with sensitivity analyses excluding major protocol deviations. Patients without CR or PR were considered treatment failures. Subgroup analyses were descriptive only and not intended to support definitive efficacy conclusions.

Supplementary Figures

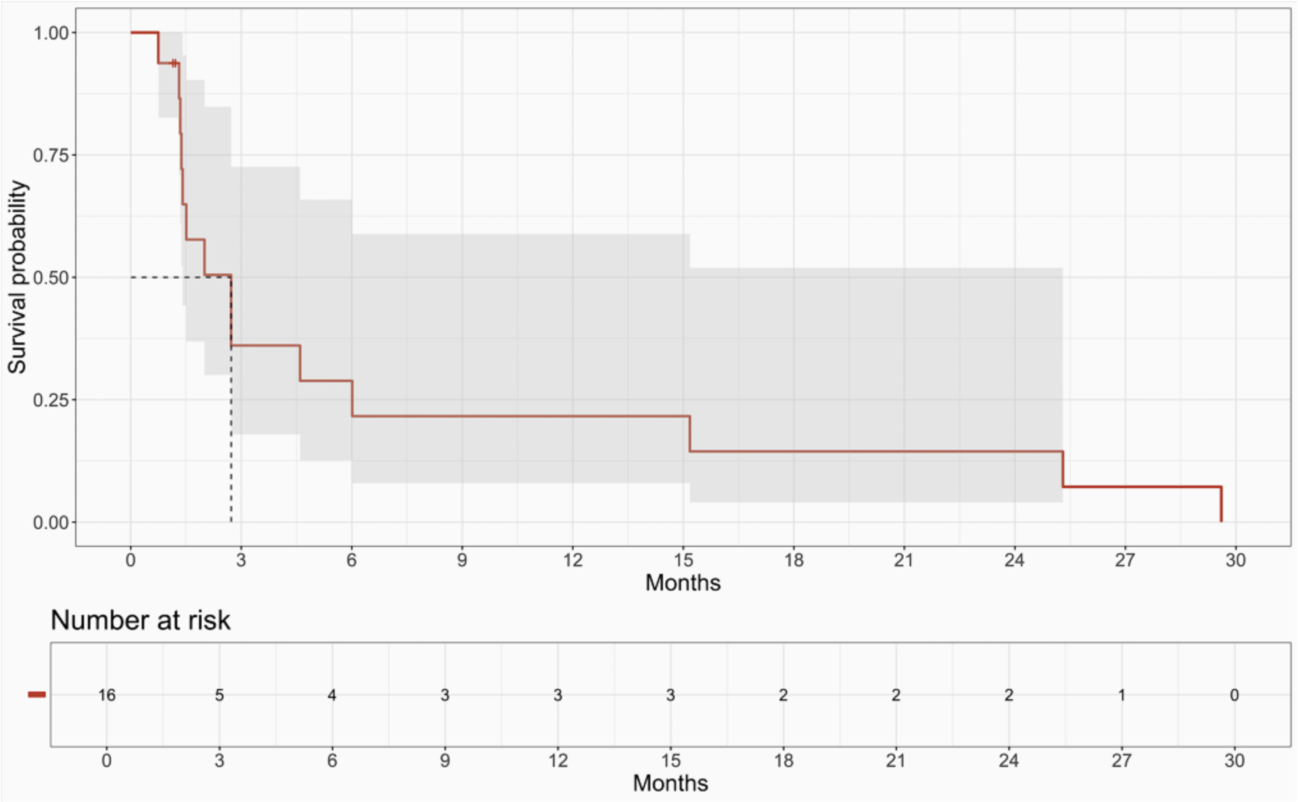

Figure 1. Kaplan-Meier curve depicting progression-free survival for all evaluable patients (N=16). Shaded area represents the 95% confidence interval. Median PFS was 2.7 months. Numbers below the x-axis indicate patients at risk at each time point.

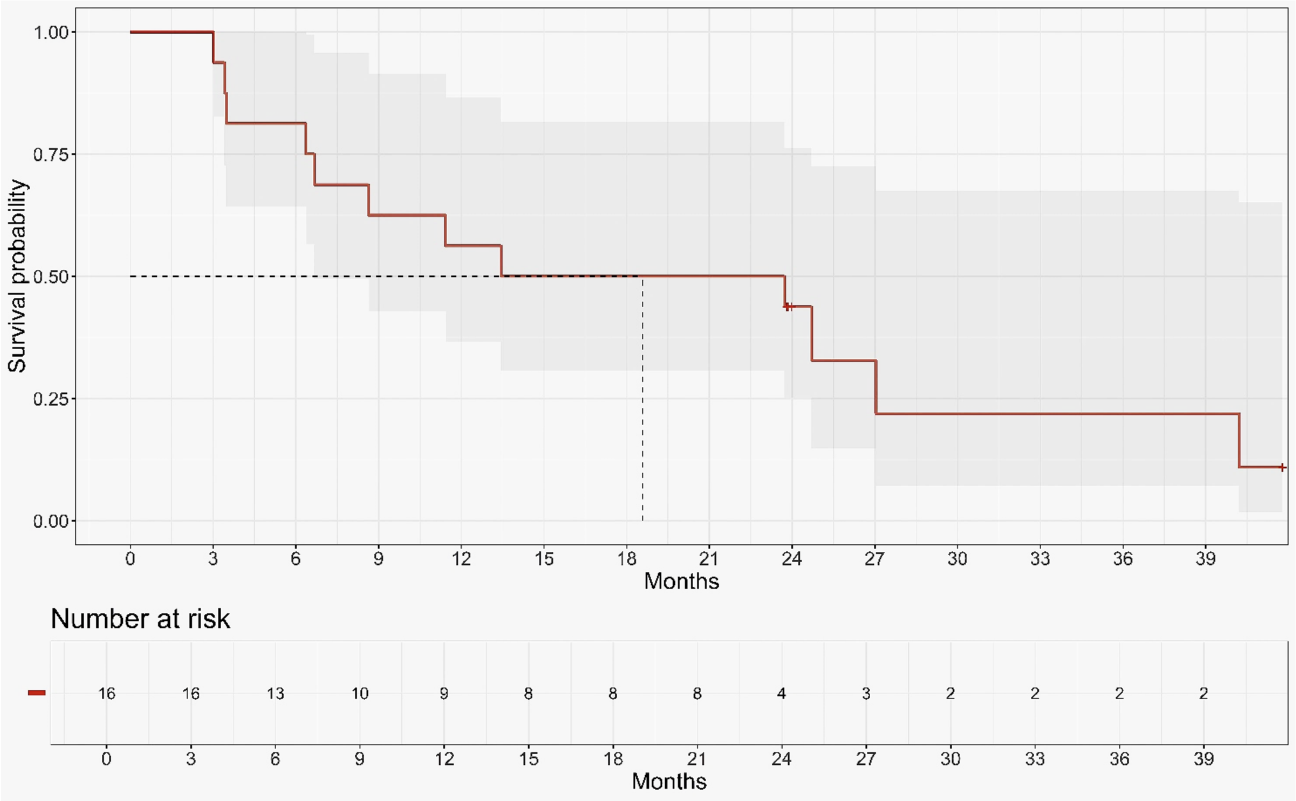

Figure 2. Kaplan-Meier curve depicting overall survival for all evaluable patients (N=16). Shaded area represents the 95% confidence interval. Median OS was 18.6 months. Numbers below the x-axis indicate patients at risk at each time point.

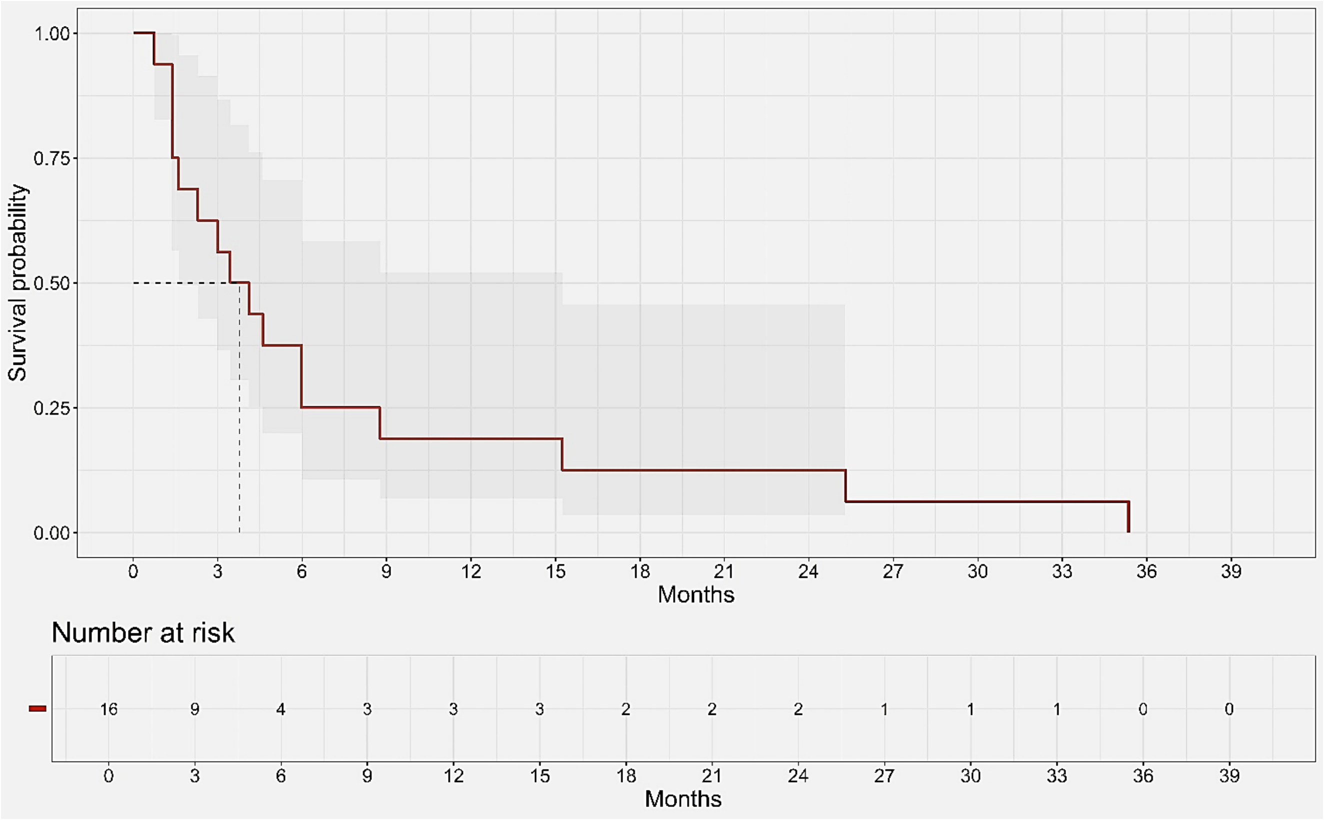

Figure 3. Treatment duration. Red line indicates time on treatment for all evaluable patients (N=16). Shaded area represents the 95% confidence interval. Median treatment duration was 3.8 months. Numbers below the x-axis indicate patients remaining on treatment at each time point.

Supplementary Table

Table 1. General Toxicity Profile

Treatment-Related Adverse Event	All Grades n (%)	Grade 3–4 n (%)
**ALT increase**	11 (69%)	3 (19%)
**AST increase**	10 (63%)	3 (19%)
**Fatigue**	10 (63%)	3 (19%)
**Nausea**	7 (44%)	0
**Thrombocytopenia**	7 (44%)	1 (6%)
**Hyponatremia**	6 (38%)	1 (6%)
**Diarrhea**	5 (31%)	0
**Hypophosphatemia**	5 (31%)	0
**Hypothyroidism**	5 (31%)	0
**Leukopenia**	5 (31%)	0
**Anorexia**	4 (25%)	0
**Headache**	4 (25%)	0
**Hypertension**	4 (25%)	2 (13%)
**Lipase increase**	4 (25%)	1 (6%)
**Mucositis (oral)**	4 (25%)	0
**Vomiting**	4 (25%)	0
**Weight loss**	4 (25%)	0
**Alkaline phosphatase increase**	3 (19%)	0
**Anemia**	3 (19%)	0
**Hyperbilirubinemia**	3 (19%)	1 (6%)
**Creatinine increase**	3 (19%)	0
**Hyperthyroidism**	3 (19%)	0
**Hypoalbuminemia**	3 (19%)	0
**Lymphopenia**	3 (19%)	1 (6%)
**Neutropenia**	3 (19%)	0
**Pruritus**	3 (19%)	0
**Bloating**	2 (13%)	0
**Chills**	2 (13%)	0
**Dry mouth**	2 (13%)	0
**Dry skin**	2 (13%)	0
**Dysgeusia**	2 (13%)	0
**Fever**	2 (13%)	0
**Flu-like symptoms**	2 (13%)	0
**Muscle weakness**	2 (13%)	1 (6%)
**Hepatic failure**	2 (13%)	2 (13%)
**Hoarseness**	2 (13%)	0
**Hypokalemia**	2 (13%)	0

**Hypomagnesemia**	2 (13%)	0
**Hypotension**	2 (13%)	0
**Muscle cramp**	2 (13%)	0
**Oral pain**	2 (13%)	0
**Rash (maculopapular)**	2 (13%)	0
**Amylase increase**	2 (13%)	0
**Sinus tachycardia**	2 (13%)	0
**TSH increase**	2 (13%)	0
**Abdominal distension**	1 (6%)	0
**aPTT prolonged**	1 (6%)	0
**Adrenal insufficiency**	1 (6%)	0
**Alopecia**	1 (6%)	0
**Appendicitis**	1 (6%)	1 (6%)
**Arthralgia**	1 (6%)	0
**Back pain**	1 (6%)	0
**LDH increase**	1 (6%)	0
**Bruising**	1 (6%)	0
**Constipation**	1 (6%)	0
**Cough**	1 (6%)	0
**Dry eye**	1 (6%)	0
**Dysphagia**	1 (6%)	0
**Peripheral edema**	1 (6%)	0
**Fall**	1 (6%)	0
**Fibrinogen decreased**	1 (6%)	1 (6%)
**Flatulence**	1 (6%)	0
**GGT increase**	1 (6%)	0
**Gum infection**	1 (6%)	0
**Hearing impaired**	1 (6%)	1 (6%)
**Hemorrhoids**	1 (6%)	0
**Hyperglycemia**	1 (6%)	1 (6%)
**Hyperhidrosis**	1 (6%)	0
**Hypersomnia**	1 (6%)	0
**Hypocalcemia**	1 (6%)	0
**Infusion reaction**	1 (6%)	0
**Laryngeal mucositis**	1 (6%)	0
**Myalgia**	1 (6%)	1 (6%)
**Nail changes**	1 (6%)	0
**Chest pain (non-cardiac)**	1 (6%)	0
**Oral hemorrhage**	1 (6%)	0
**Pain**	1 (6%)	1 (6%)
**Pericardial effusion**	1 (6%)	1 (6%)

**Productive cough**	1 (6%)	0
**Proteinuria**	1 (6%)	0
**Sinus bradycardia**	1 (6%)	0
**Sore throat**	1 (6%)	0
**Thromboembolic event**	1 (6%)	1 (6%)
**Wound complication**	1 (6%)	0
**Jaw pain**	1 (6%)	0
